# Mechanocatalytic Hydrogenolysis of the Lignin Model Dimer Benzyl Phenyl Ether over Supported Palladium Catalysts

**DOI:** 10.1021/acssuschemeng.4c03590

**Published:** 2024-08-05

**Authors:** Erin V. Phillips, Andrew W. Tricker, Eli Stavitski, Marta Hatzell, Carsten Sievers

**Affiliations:** † School of Chemistry and Biochemistry, Georgia Institute of Technology, Atlanta, Georgia 30332, United States; ‡ Independent Researcher, Washington, D.C. 20009, United States; § National Synchrotron Light Source II, Brookhaven National Laboratory, Upton, New York 11973, United States; ∥ School of Chemical and Biomolecular Engineering, Georgia Institute of Technology, Atlanta, Georgia 30332, United States; ⊥ George W. Woodruff School of Mechanical Engineering, Atlanta, Georgia 30318, United States

**Keywords:** ball mill, biomass conversion, depolymerization, heterogeneous catalysis, lignin model compounds, mechanochemistry

## Abstract

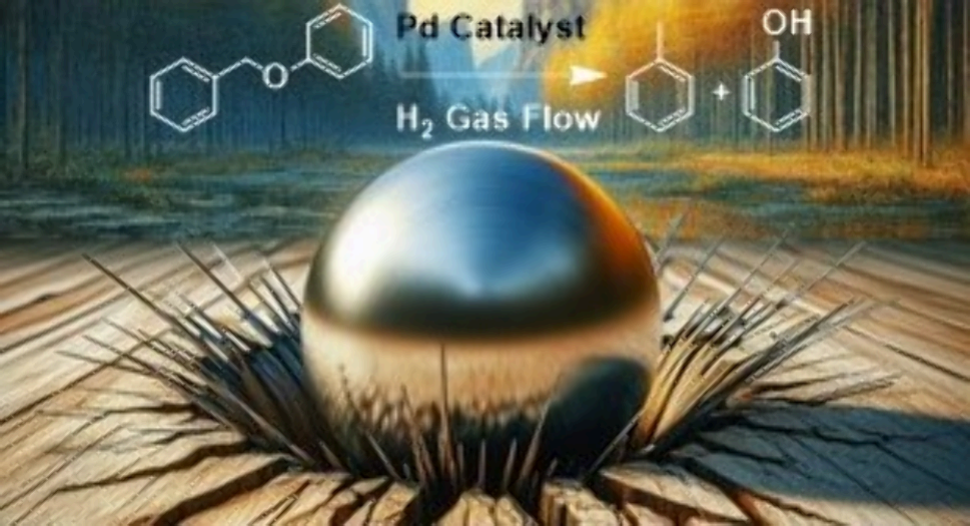

This work demonstrates the mechanocatalytic hydrogenolysis of the ether bond in the lignin model compound benzyl phenyl ether (BPE) and hardwood lignin isolated by hydrolysis with supercritical water. Pd catalysts with 4 wt % loading on Al_2_O_3_ and SiO_2_ supports achieve 100% conversion of BPE with a toluene production rate of (2.6–2.9) × 10^–5^ mol·min^–1^. The formation of palladium hydrides under H_2_ gas flow contributes to an increase in the turnover frequency by a factor of up to 300 compared to Ni on silica–alumina. While a near-quantitative toluene yield is obtained, some of the phenolic products remain adsorbed on the catalyst.

## Abstract 2

Hydrogenolysis of ether bonds in lignin is achieved by ball milling with supported palladium catalysts that readily form bulk hydrides.

Lignin, a major component of lignocellulosic biomass, is the most abundant source of aromatics in nature but remains significantly underutilized due to lignin’s complex and amorphous structure.^1−5^ The three-dimensional polymer is composed of substituted phenolic monomers, called monolignols, that contain a variable number of hydroxy and methoxy groups and a propyl side chain. A variety of ether, alkyl, or aryl bonds connect the monomers. These bonds are difficult to selectively cleave, resulting in the recalcitrant and chemically diverse structure of lignin. Various approaches, such as pyrolysis and reductive catalytic fractionation (RCF), have been used to depolymerize lignin to its aromatic components, but each method presents a unique set of complications. Pyrolysis is energy-intensive due to the high temperatures required, while RCF can only be applied to unfractionated lignocellulosic biomass and is often associated with coking issues and the repolymerization of intermediates to unreactive polyaromatics.^6−11^ Due to incomplete bond cleavage and repolymerization, monomer yields resulting from RCF typically do not exceed ∼55% even under optimized conditions.^12^ Hydrogenolysis specifically targets the cleavage of carbon–carbon or carbon–heteroatom bonds, followed by stabilization via the addition of an H atom.^6−8^ Homogenous catalysts can effectively cleave ether bonds in lignin, but difficult separations and low product purity are common disadvantages.^8^ Alternatively, solid catalysts, such as supported Ni, Pd, or Pt, allow for easier separation under reasonably mild conditions, but contacting them with a solid feedstock like lignin that is insoluble in most common solvents is a significant challenge.^7,8,12−14^ Pd was specifically chosen as the primary active metal for this study due to its ability to form interstitial hydrides as a hydrogen storage reservoir, which is advantageous in facilitating hydrogenolysis reactions.^15−17^

Mechanocatalysis is an emerging approach that utilizes either the vibrational or rotational forces in a ball mill or similar device to drive reactions in a solvent-free environment.^18−23^ Compared to other traditional methods for lignin conversion, such as pyrolysis, mechanocatalysis is an attractive alternative because no additional heating or pressurization is required to complete many reactions.^19,20,24,25^ A variety of phenomena have been considered as potential drivers for mechanochemical reactions including the formation of thermal hot spots upon impact between the balls and vessel walls, highly active transient sites on a mechanically excited catalyst surface, and increased solid–solid contact between the solid feedstock and catalyst particles as a result of consistent mixing and shearing.^19,26,27^

Several studies of mechanocatalysis as an approach to depolymerizing lignocellulosic biomass and lignin model compounds have been conducted.^5,14,28−43^ Rinaldi et al. demonstrated the depolymerization of both cellulose and biomass to water-soluble sugars and furfurals with complete conversion and yields of water-soluble oligosaccharides and lignin oligomers exceeding 90%.^28−33^ Bolm’s works have examined the conversion of lignin model compounds representing β-O-4 linkages in base-catalyzed^35^ and oxidative reactions.^37^ The base-catalyzed reactions produced monomer yields of up to 94%, while the oxidative transformation resulted in aryl-c_α_ cleavage of the β-O-4 model, resulting in quinone (91% yield) and guaiacol (82% yield) as the two main products. Sievers et al. recently demonstrated hydrogenolysis of the α-O-4 bond in the lignin model compound, benzyl phenyl ether (BPE), over supported Ni catalysts.^43^ Due to the similar bond strengths, this ether bond can be considered representative of the β-O-4 linkages found in lignin. In this study, H_2_ was continuously flowed through a modified vessel to avoid using a more expensive solid hydrogen source such as NaBH_4_.^44−46^ The study showed full BPE conversion using a commercial Ni (53 wt %) catalyst supported on a silica–alumina support (Ni_53_/Si–Al) within 3 h and a turnover frequency (TOF) of 0.001 min^–1^. The present study advances this previously established process by utilizing supported Pd catalysts with improved TOFs up to 0.342 min^–1^(Figure S3).

The Pd loading of the catalysts was determined by inductively coupled plasma to be 4 wt %, and the catalysts were denoted as Pd_04_/Al_2_O_3_ and Pd_04_/SiO_2_. Transmission electron microscopy (TEM) imaging (Figure S4) showed an average Pd particle size of 3.5 nm for Pd_04_/Al_2_O_3_ and 7.3 nm for Pd_04_/SiO_2_ before milling, respectively. By comparison, CO chemisorption analysis gave particle sizes of 2.5 nm for Pd_04_/Al_2_O_3_ and 4.7 nm for Pd_04_/SiO_2_ (Section S2.5). The calculated dispersion of Pd using CO chemisorption was 45.5% for Pd_04_/Al_2_O_3_ and 23.7% for Pd_04_/SiO_2_, while the dispersion values based on TEM were 32.3% and 15.4%. Further characterization of the fresh catalysts can be found in the Supporting Information (SI) including X-ray diffraction (XRD; Section S2.9) and N_2_ physisorption (Figure 4).

BPE was milled in combination with one of the catalysts using a Retsch MM400 instrument in a stainless-steel vessel under H_2_ flow at atmospheric pressure. Volatile products were collected in a methanol trap during milling while nonvolatile products were quantified by washing the catalyst–feedstock mixture after milling. Gas chromatography with flame ionization detection was used to determine all yields, conversions, and carbon efficiencies. The main two products from hydrogenolysis at the α-carbon and oxygen were toluene and phenol (Scheme 1). The reaction path of mechanocatalytic BPE ether bond cleavage appears to be similar to the one previously shown for the equivalent thermochemical reaction over supported Pd catalysts.^47,48^ Hydrogenation of phenol yielded cyclohexanol as a minor product (Section S1.3). We did not observe the hydrogenation of toluene because constant purging allowed toluene to exit the milling vessel before a secondary reaction could occur. Pd_04_/SiO_2_ and Pd_04_/Al_2_O_3_ behaved similarly under these conditions. Toluene was produced at a rate of 2.61 and 2.91 mol·min^–1^, respectively, while phenol yields averaged 48% after 1 h of reaction (Figure 1). By comparison, Ni_53_/Si–Al produced toluene at a rate of 1.01 mol·min^–1^ over 3 h, with 42% cyclohexanol yield and 17% phenol yield, and the undesired secondary hydrogenation of phenol to cyclohexanol was more prominent.^43^ The overall carbon balance of the reaction ranged from 75 to 80% for all three catalysts, and full conversion was observed.

**Scheme 1:**
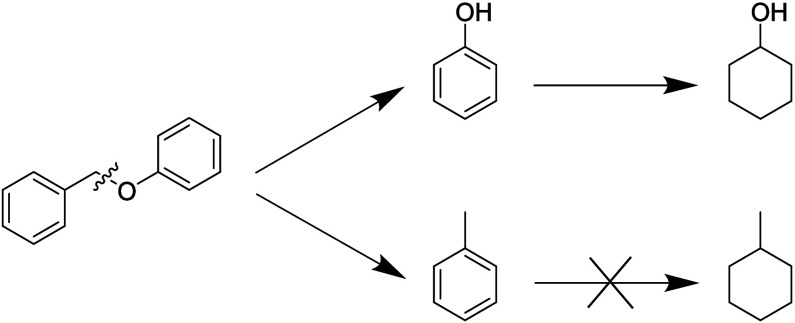
Reaction Scheme Depicting the Cleavage of BPE Producing Phenol and Toluene Followed by Hydrogenated Products Cyclohexanol and Methylcyclohexane

**Figure 1:**
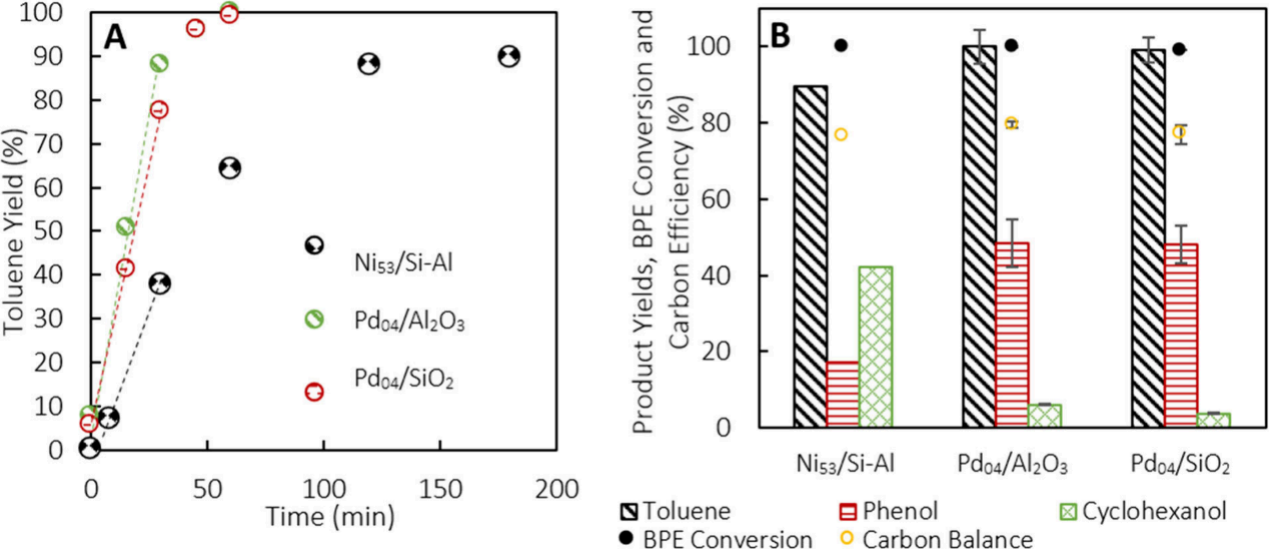
(A) Toluene yields over Ni_53_/Si–Al, Pd_04_/SiO_2_, and Pd_04_/Al_2_O_3_ over 1–3 h of milling and (B) toluene, phenol, cyclohexanol yields, CE, and BPE conversion after the first run. A total of 0.25 g of Pd_04_ catalysts was used at 20 Hz with 0.2 g of BPE and 10 × 10 mm grinding balls at 15 sccm H_2_ flow. Alternatively, 1.00 g of Ni_53_/Si–Al was used with 2 mm × 15 mm balls.

Thermogravimetric analysis (TGA) in air showed a mass loss of 16 wt % for both catalysts, indicating that the unaccounted for carbon was present as an adsorbed species on the catalyst (Section S2.8). Because the deficit in the mass balance aligned well with the mass of carbonaceous deposits on the catalyst, it is suggested that the phenolic moiety from BPE remained chemisorbed on the alumina and silica supports as phenolates, which has been reported for similar catalysts in thermochemical reactions.^49,50^ Because Lewis acid sites are the preferred binding sites for these species, pyridine adsorption followed by IR spectroscopy was used to determine the concentration of these sites. For the Pd_04_/Al_2_O_3_ catalyst, no Brønsted acid sites were found, while the concentration of Lewis acid sites decreased from 120 μmol·g^–1^ in the fresh catalyst to 1.5 μmol·g^–1^ in the spent one, indicating that the phenolic products saturated the Lewis acid sites on the support (Figures S7 and S8). The phenolic compounds that did not bind after this saturation were easily removed, which resulted in a phenol yield of 48%; minor cyclohexanol yields of 4–6% were also observed due to the hydrogenation of nonadsorbed phenols. While silica supports are generally considered inert, it has been shown that chemisorption of alcohols readily occurs at defect sites that are formed when silica ruptures during milling (Figure S11).^51−53^ In addition to chemisorbed phenolates on such defect sites, phenol can physisorb onto silanol sites.^54−56^ The physisorbed phenols are more easily removed and comprise most of the phenol yield that was observed after washing. This ultimately suggests that the Lewis acid site concentration plays a minor role in absorption. Lewis acid site occupation accounts for roughly 5% of the unaccounted for phenol for Pd_04_/Al_2_O_3_, while defects account for 95%. Pd_04_/SiO_2_ silanol sites and defect sites account for 100% of the missing phenol, considering that the Lewis acid site concentration initially was only 0.012 μmol·g^–1^. Further calculations are shown in Section S2.7.

Compared to the Ni_53_/Si–Al catalyst, which had a TOF of 0.001 min^–1^, the TOFs for Pd_04_/Al_2_O_3_ and Pd_04_/SiO_2_ were 0.342 and 0.289 min^–1^, respectively. We hypothesized that this increase in the TOF is due to the ability of Pd to form interstitial hydrides. These immobilized hydrogen species are expected to be more readily available for reactions during the impact of a ball because they cannot escape to the sides like gaseous H_2_ would. Interstitial hydride formation has been extensively used for Pd membranes for H_2_ separation.^15−17,57,58^ At the surface of Pd membranes or particles, H_2_ is able to dissociate into H atoms, which allows hydrogen to easily diffuse into the Pd particle.^16,17,59^ These singular H atoms can then be combined with other molecules or compounds on the Pd surface. Many other metals, such a Pt^60^ and Ti,^61^ can form surface hydrides, but facile hydrogen diffusion into the particle is unique to Pd. Nonconstant changes in the morphology of the Pd particles during milling help to expose reactive hydrides, which are readily available to participate in the hydrogenolysis reaction. The presence of this hydrogen reservoir^16^ is suggested to significantly increase the efficiency of BPE conversion.

The expected presence of interstitial hydrides was probed by X-ray absorption spectroscopy (XAS; Figure 2).^62^ The spectra were dominated by metallic Pd (2.5 Å in *R* space), but a contribution of PdO (1.5 Å in *R* space) was clearly visible.^63,64^ The spectra of the samples milled in hydrogen showed a decreased contribution of PdO, while the primary peak for Pd–Pd bonds at 2.5 Å increased in intensity for Pd/Al_2_O_3_ and slightly shifted to a higher bond distance for both catalysts from 2.54 to 2.57 Å. The intensity shifts indicated that hydrogen milling created a sufficiently reducing environment to reduce any oxidized Pd in situ; for Pd_04_/SiO_2_, the Pd present in the fresh sample had a lower PdO concentration, which is why a significant increase in the peak intensity - was not observed, but a slight hydride shift still occurred. This reduction occurred in tandem with the hydrogenolysis reaction, which was promoted by reduced Pd. The rightward shift in the Pd–Pd peak was indicative of hydride formation, which resulted in a slight expansion of the Pd lattice.^16,17,59,63,65,66^

**Figure 2:**
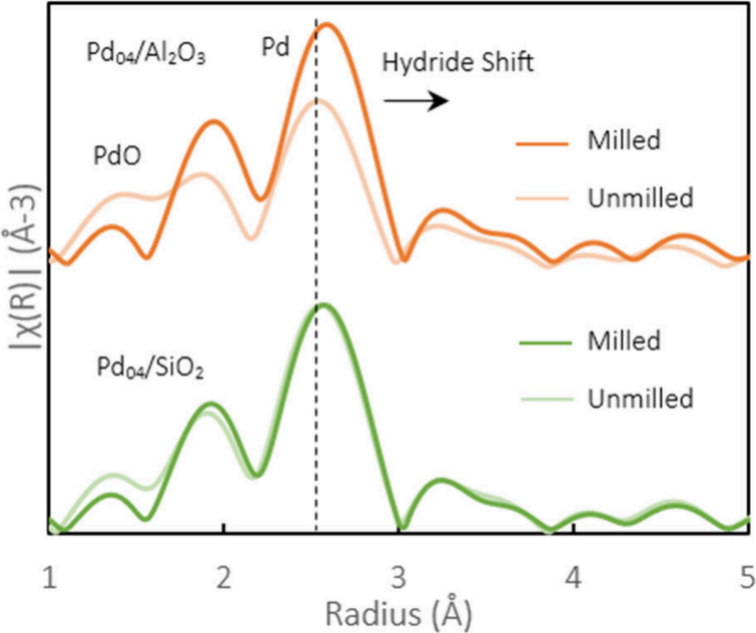
Fourier-transformed *R* space of the experimental Pd K-edge EXAFS signals for Pd_04_/Al_2_O_3_ and Pd_04_/SiO_2_ catalysts before and after milling under H_2_ to depict hydride formation.

The recyclability of Pd_04_/Al_2_O_3_ and Pd_04_/SiO_2_ was tested after calcining the spent catalysts to remove organic residues (Figure 3). Although calcination results in the oxidation of Pd to PdO, it had to be verified that milling under H_2_ could restore the reduced Pd particles. EXAFS analysis of spent catalysts after calcination showed a significant increase in the intensity of PdO (1.5 Å) coupled with a reduction in the Pd–Pd bond length (2.5 Å) (Figure S6). After the hydrogenolysis reaction with the recycled catalysts, a shift back to reduced Pd coupled with palladium hydride formation was observed, indicating that milling under H_2_ gas flow was able to sufficiently reduce the fresh and recycled Pd catalysts.

**Figure 3:**
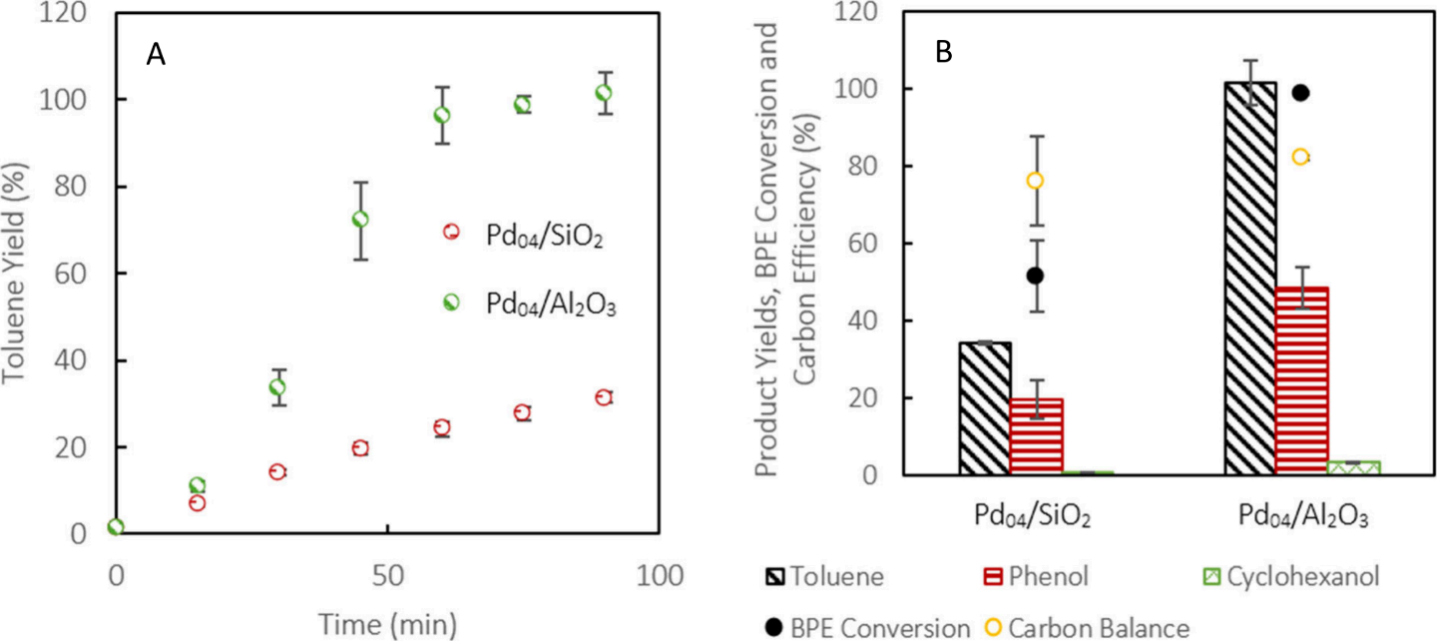
(A) Toluene yields over recycled Pd_04_/SiO_2_ and Pd_04_/Al_2_O_3_ over 90 min of milling and (B) total toluene, phenol, cyclohexanol yields, carbon balance, and BPE conversion of recyclability tests for Pd_04_/Al_2_O_3_ and Pd_04_/SiO_2_.

With calcination, 88% of Pd_04_/SiO_2_ and 82% of Pd_04_/Al_2_O_3_ catalysts were recovered on average. To determine the catalytic reactivity after calcination, trials were conducted using a combination of recycled and fresh catalysts. To maintain consistency, 0.20 g of the calcined catalyst was combined with 0.05 g of fresh catalyst, resulting in a 4:1 ratio (Figure 3). With Pd_04_/SiO_2_, the maximum toluene yield reached was 34% after 90 min. By comparison, full conversion and a 100% toluene yield was observed with Pd_04_/Al_2_O_3_. Phenol production over Pd_04_/Al_2_O_3_ was 49%, which was slightly higher than the initial phenol yield over Pd_04_/Al_2_O_3_, while the Pd_04_/SiO_2_ yield was much lower, resulting in only 20% (Figure 3B).

Reduced activity of the catalyst can be attributed to a reduced surface area (SA), increased Pd particle size, and alteration of the support throughout the milling process. Initially, Pd_04_/SiO_2_ and Pd_04_/Al_2_O_3_ had SAs of 209 and 106 m^2^·g^–1^, respectively (Figure 4). After initial milling, the SA of Pd_04_/SiO_2_ reduced to 76 m^2^·g^–1^, which increased to 101 m^2^·g^–1^ after calcination. Upon recycling, the catalyst became denser and the SA reduced to 12 m^2^·g^–1^ after milling. The SA of Pd_04_/Al_2_O_3_ was drastically reduced to 3 m^2^·g^–1^ after the first reaction but increased to 116 m^2^·g^–1^ after calcination, slightly exceeding the initial value. This partially explains why Pd_04_/Al_2_O_3_ was more effective when recycled in comparison to Pd_04_/SiO_2_. TEM images after milling showed the agglomeration of Pd particles (Figure S4). The Pd particles remained active but were less effective compared with the fresh counterparts due to reduced dispersion. For Pd_04_/Al_2_O_3_ specifically, amorphization of the Al_2_O_3_ support was also observed via XRD (Figure S10). Changes in the SiO_2_ support structure were more effectively seen with ATR FTIR spectroscopy (Figure S11). The spectra showed the formation of silanol sites and both chemi- and physisorbed interactions between oxygenated products and mechanically activated SiO_2_. The combination of these characteristics elucidates how the degradation of these catalysts throughout the milling process affects the recyclability potentials of both Pd_04_/SiO_2_ and Pd_04_/Al_2_O_3_.

**Figure 4:**
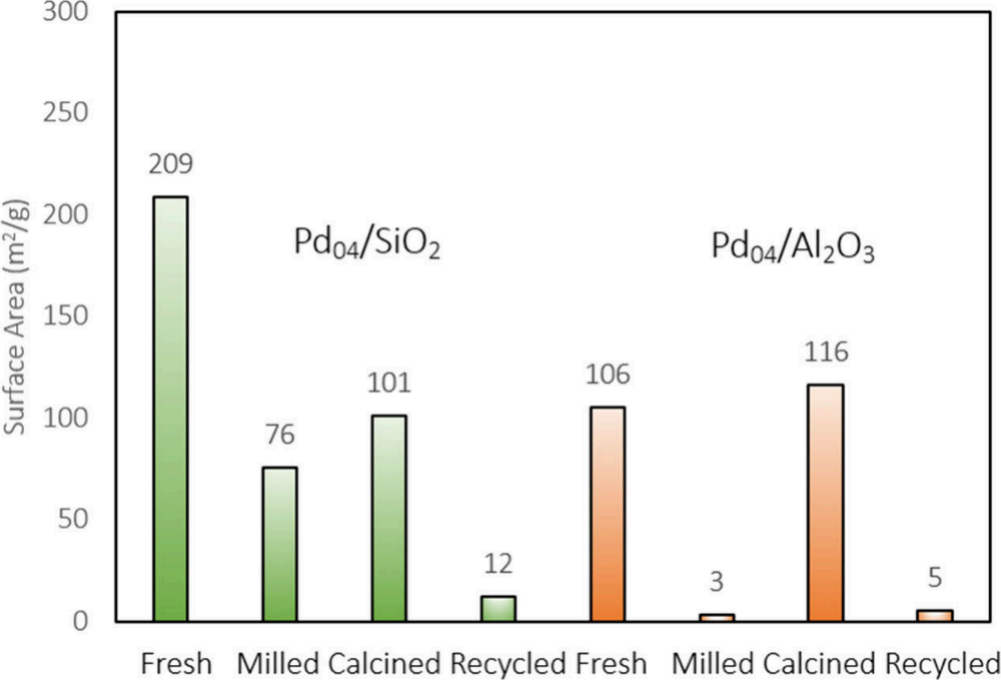
SA analysis of Pd_04_/SiO_2_ and Pd_04_/Al_2_O_3_ (m^2^·g^–1^).

To illustrate the potential application of mechanocatalytic hydrogenolysis over Pd-based catalysts beyond model compounds, the conversion of supercritical-water-extracted lignin was studied under identical milling conditions. Gel permeation chromatography (GPC) showed significant changes in the average *M*_w_ after 1 h of milling (Figure 5). The unmilled lignin showed a significant amount of lignin fragments ranging from 1000 to 30000 g·mol^–1^ (10^3^–10^4.5^). There were two main peaks, which equate to roughly 430 and 660 g·mol^–1^. The monolignols *p*-coumaryl, coniferyl, and sinapyl alcohol have molar masses of 150, 180, and 210 g·mol^–1^, respectively. Thus, the two peaks are attributed to dimers and trimers, respectively. After milling, the abundance of products with intermediate masses (i.e., 1800–30000 g·mol^–1^) decreased markedly, while the amount of monolignol dimers and trimers increased strongly and small amounts of larger products were formed. As illustrated by the hydrogenolysis of ether bonds in BPE, the reduction in *M*_w_ was likely a result of the hydrogenolysis of α-O-4 and β-O-4 bonds. To test this hypothesis, heteronuclear single quantum coherence (HSQC) NMR spectroscopy was conducted to analyze the change in the linkage composition of the supercritical-water-extracted lignin samples that were milled for 1 h using Pd_04_/Al_2_O_3_ and Pd_04_/SiO_2_ under the same conditions as BPE. The results (Section S2.11) demonstrate that the abundance of the β-O-4_β_ linkage was reduced by 5.4% using Pd_04_/Al_2_O_3_ and 12.5% for Pd_04_/SiO_2_, using the ββ_β_ peak as a standard. ββ_β_ bond integration was assumed to be unaffected by the catalysts in a hydrogenolysis environment, where the integral of the peak remained constant before and after milling. This ultimately shows that this approach is feasible with real lignin, and with additional optimization and longer milling conditions, lignin could be converted with a high depolymerization efficacy.

**Figure 5:**
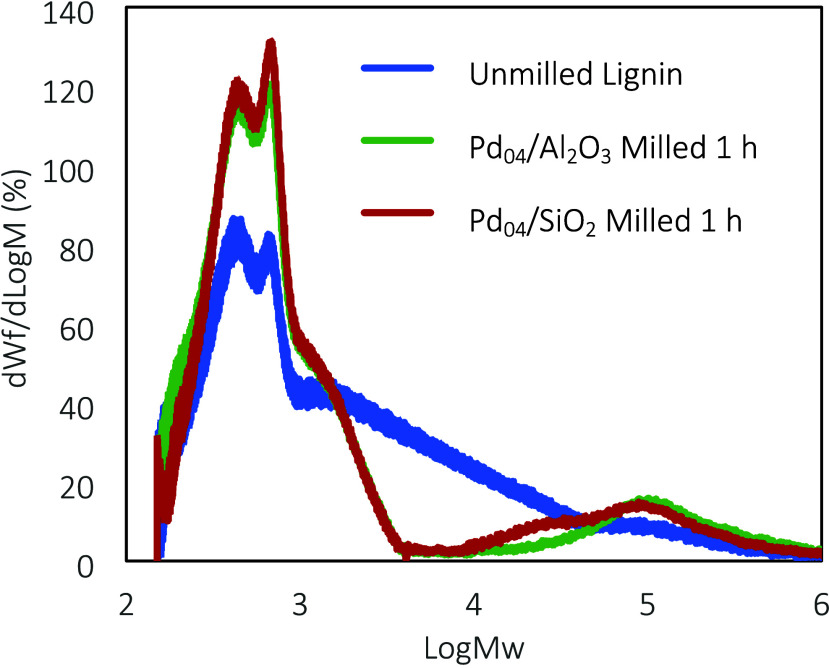
Molecular weight distributions of milled and unmilled lignin determined by GPC.

In summary, this work demonstrates the potential of using Pd-based catalysts for the hydrogenolysis of lignin in a mechanocatalytic environment. Using Pd as the primary metal in these heterogeneous catalysts provided superior activity compared to that of Ni-based catalysts used under the same conditions. The ability of Pd to form interstitial hydrides that readily participate in hydrogenolysis reactions enhances the rate of successful collisions. Chemisorption of phenolates has been identified as a remaining challenge of this process.

## Supporting Information Available

Full experimental details and analytical data including TOF and particle percent dispersion calculations, TEM images, XANES/EXAFS spectra, XRD, pyridine adsorption concentrations along with FTIR spectra, TGA, FTIR analysis conducted using an ATR attachment, and 2D HSQC NMR of the lignin samples before and after milling (PDF)
