# Supplementary material for: Mechanocatalytic Hydrogenolysis of the Lignin Model Dimer Benzyl Phenyl Ether over Supported Palladium Catalysts
Source: ACS Sustain Chem Eng. 2024 Aug 5;12(33):12306–12. doi: 10.1021/acssuschemeng.4c03590 (PMC11337168; doi:10.1021/acssuschemeng.4c03590)
Supplement: Supplementary file 1 — sc4c03590_si_001.pdf [file sc4c03590_si_001.pdf]

Supporting Information

Mechanocatalytic Hydrogenolysis of the Lignin Model Dimer Benzyl Phenyl Ether over Palladium-Supported Catalysts

Erin V. Phillips[a], Andrew W. Tricker[b], Eli Stavitski[c], Marta Hatzell[d,e], Carsten Sievers[a,d,*]

[a]School of Chemistry and Biochemistry, Georgia Institute of Technology, Atlanta, GA 30332, United States

[b]Independent Researcher, Washington, DC 20009, United States

[c]National Synchrotron Light Source II, Brookhaven National Laboratory, Upton, New York 11973, United States

[d]School of Chemical & Biomolecular Engineering, Georgia Institute of Technology, Atlanta, GA 30332, United States

[e]George W. Woodruff School of Mechanical Engineering, Atlanta, GA 30318, United States

Email: carsten.sievers@chbe.gatech.edu

Number of Pages: 24

Number of Figures: 13

Number of Tables: 4

Section S1: Experimental and Analytical Procedures

S1.1 Catalytic Reactions and Product Quantification

S1.1.1 Chemicals

Benzyl phenyl ether (BPE, 97%) was purchased from Alfa Aesar. O-xylene (>99.0%) was obtained from Sigma Aldrich, along with Pd on Al_2O_3. Pd on SiO_2 was purchased from STREM Chemicals, pre-reduced. The two catalysts were commercially advertised as having a 5 wt% loading, but ICP was conducted to verify the actual Pd loading. Methanol (ACS grade) was purchased from VWR while H_2 and N_2 (UHP, Grade 5) were purchased from Airgas.

S1.1.2 (A) Mechanocatalytic Hydrogenolysis Reactions

The mechanocatalytic reactions were conducted using a Retsch MM400 vibrational ball mill. A 25 mL stainless steel vessel was used to hold the feedstock and catalyst during the reactions, which was adapted with two outlets to allow for continuous gas flow. All reactions were run at a frequency of 20 Hz with 10 stainless steel balls which measured 10 mm in diameter. A scheme and image for the reaction setup is depicted below in Figure S1. It is notable to mention that this vessel setup is similar to the vessel designed by the Schüth group.[1]

A 25 mL methanol trap was placed at the end of the effluent line to collect all volatile products from the reactor. The line, also composed of stainless steel, measured 1/8 inch in diameter and was wrapped in heating tape to ~ 150°C to ensure that all volatile products eluted through the line. The vessel itself was not heated, but it is estimated that milling caused a temperature increase of approximately 30 – 40°C as a result of stainless-steel collision impact. The methanol trap was placed in an ice water bath and cooled to ~4°C to guarantee that the methanol would not evaporate with the volatile products throughout the milling process. The lines directly connected to the inlet and outlet of the vessel were made of Teflon and measured 1/8 inch in diameter. A ¼ inch frit was placed in the Swagelok connection at the outlet to keep any catalyst from clogging the gas lines and leaving the vessel during milling.

For each experiment, 0.2000 ± 0.0005 g of BPE and 0.2500 ± 0.0005 g Pd catalyst were added to the vessel, while ~ 0.030 – 0.050 g of o-xylene was added to the methanol trap as an internal standard. Once the vessel was closed and connected to the gas flow lines, the reaction began by milling at 3 Hz under N_2 at 15 sccm for 15 minutes, to purge the vessel of atmospheric air, followed by purging for 15 min of H_2 flow at 15 sccm and 3 Hz. The mill was then run for 60 min at 20 Hz under 15 sccm H_2, followed by 30 min of stand-still N_2 purging, to make sure that all volatile products had been removed. Gas flow was controlled using an Omega mass flow controller (MFC).

To measure toluene production evolution, ~0.5 mL aliquot samples were taken from the methanol trap every 15 mins for the first 60 mins, and once again after the final N_2 purge. After reaction completion, the contents of the vessel were collected and measured; on average, 80 – 90% of the catalyst and product was able to be collected, while the remaining 10 – 20% remained adhered to the balls, the sides of the vessel walls or compacted into the inlet and outlet connections. The collected catalyst-product mixture was diluted with 10 – 15 mL of methanol, and 0.030 g to 0.050 g of o-xylene was once again added as an internal standard. The mixture was sonicated for 30 mins and filtered afterwards to obtain a pure liquid sample of the diluted non-volatile products for product quantification using GC-FID.

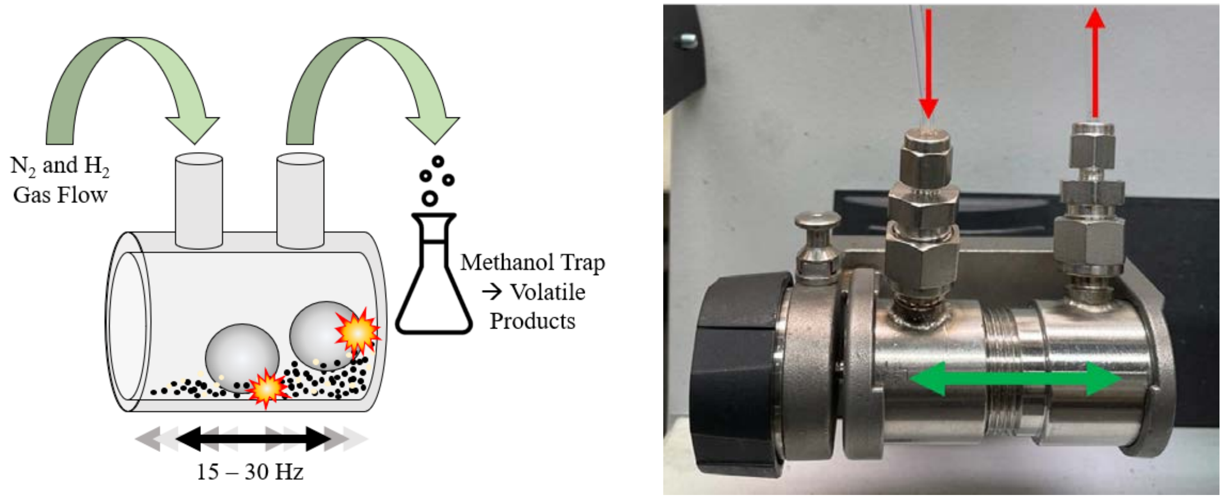

Figure S1: Scheme of the milling setup (left) using a Retsch MM400 with adaptations for gas flow through the vessel. A picture of the milling vessel is shown to the right.

1.1.2 (B) Calcination followed by Recyclability Reactions

Calcination trials were conducted for Pd_{04}/Al_2O_3 and Pd_{04}/SiO_2. After initial milling, the catalyst-product mixture was collected from the vessel and weighed. Instead of non-volatile product analysis though, the mixture was heated in a tube furnace; the temperature was ramped to 450°C at 10°C/min and held at 450°C for 2 h under house air. After cooling, the catalyst was weighted to determine the loss in weight of non-volatile products and overall catalyst recovery. On average, 0.2150 g of Pd_{04}/Al_2O_3 was recollected while 0.2300 g was recovered for Pd_{04}/SiO_2. To conduct the recyclability reaction, all parameters were held the same with the exception that 0.20 of recycled catalyst was measured while 0.05 of fresh catalyst was measured. With these trials, all gas flow also remained the same, but the mill was run for 90 mins instead of 60 mins. Toluene collection and non-volatile product collection was conducted in the same manner as before.

S1.1.3 Gas Chromatography with Flame Ionization Detection and methanizer attachment (GC-FID)

All product yields were determined using a Varian 450-GC equipped with an FID detector and PolyArc methanizer reactor (Activated Research Company). A fused silica column was used (Supelco, SPB-1, 60 m x 0.25 mm x 1.0 µm) within the GC for product separation. As previously mentioned, o-xylene was used as an internal standard, so no calibration was required when used in tandem with the PolyArc methanizer.

Analysis began upon heating the GC oven to 70°C and was held constant for 15 minutes. The temperature was then ramped to 250°C at a rate of 10°C/min and held at 250°C for 8 mins. The final ramp was to 300°C at 20°C/min, then was held for 6.5 mins before cooling began. The continuous flow rate for H_2 through the column was 10 mL/min. The split ratio for the sample injection was 1:15, and the FID analysis registered all products that exceeded a signal to noise ratio of 5:1. An auto sampler was attached to the GC-FID for the ease of completing sample analysis. Example calculations are shown below with equations S1 and S2:

$$n_{product} = \frac{A_{product}}{A_{Standard}} * \frac{C_{Standard}}{C_{Product}} * \frac{m_{Standard}}{MW_{Standard}} \quad \text{(S1)}$$

$$Yield_{Product} = \frac{n_{Product}}{n_{BPE\ Initial}} * 100\% \quad \text{(S2)}$$

$$A = GC - FID\ Peak\ Area$$

$$C = \#\ of\ carbons\ within\ the\ molecular\ structure$$

S1.1.4 Gel Permeation Chromatography (GPC)

Experiments to determine molecular weights (MW) of lignin samples were performed on a Tosoh EcoSEC Elite HLC-8420 GPC equipped with PL HFIPgel column, internal refractive index detector (RID) operating at 40°C. The eluent was 1,1,1,3,3,3-hexafluoro-2-propanol (HFIP) at a flow rate of 0.1 mL/min.

Section S1.2: Metal Sites and Morphology Techniques

S1.2.1 Inductively Coupled Plasma (ICP) Analysis

To prep the samples for ICP, the unmilled catalysts were first weighed and pre-digested in a 50 mL beaker and placed on a hot plate. 5 mL of concentrated nitric acid (Trace metal Grade from Fisher Chemical, UN2031) was added to the beaker. The mixture was heated for 30 mins at an elevated temperature, then 2.5 mL of concentrated HCl (Trace metal grade from Fisher Chemical) was added. The mixture was brought to a slight boil for an additional 30 mins, then allowed to cool to room temperature. Afterwards, the mixture was filtered through 40 ashless Whatman filter paper and washed with di-ionized water. The assembly was jointed to the filtrate, and the final volume of aqueous solution was recorded in mL. The ICP was calibrated with Pd, Si, and Al, generating a four-point calibration curve for each element of interest. The previously prepared sample was injected into the ICP, and the target elements were quantified based on the calibration. Results are shown in Table S1.

S1.2.2 Transmission Electron Microscopy (TEM)

A FEI Tecnai F30 was used to obtain TEM images at an operating kV of 300. The instrument was equipped with a thermally assisted field emission (TFE) gun, and all images were

converted and analyzed using a Gatan GIF system (Tridiem 863 UHS). A CompuStage single-tilt holder was used to hold the samples and all images are shown on a scale of 100 – 200 nm. For preparation, approximately 5 mg of the samples were sonicated in 1 mL of methanol and dispersed onto a holey carbon – copper 200 mesh grid. Initial particle sizes were calculated using the TEM images below (Figure S2) by taking an average diameter of 30 particles. ImageJ software was used for the analysis. Based on the particle size determined by TEM, the following equation (Eq S3) was used to determine the % dispersion of the particles:

$$D\% = 6 * \frac{(^{v_m}/_{a_m})}{d_{Va}} \quad \text{(S3)}$$

Here, v_m is defined as the volume occupied by an atom in the bulk of metal, where the MW of Pd is divided by the density of Pd and multiplied by Avogadro's number to give the constant value:

$$v_m = \frac{Pd_{MW}}{Pd_{Density} * AGs\ \#} = \frac{106.4\,\frac{g}{mol}}{12.03\frac{g}{cm^3} * 6.022E23}\ 1.4632\text{ E-23 cm}^3\text{/atom}$$

The variable $a_m$is the atomic cross-sectional area where 0.0787 nm^2/Pd atom is a defined, non-changing value and d_{Va} is the particle size.

S1.2.3 CO Chemisorption

CO chemisorption measurements were performed on a Mircomeritics AutoChem II 2920. Each sample was prepared by loading about 140 mg of catalyst on a bed of quartz wool in a U-shaped quartz tube. Prior to the CO chemisorption measurements, the samples were heated (at 5 °C/min) to 400 °C under 10% H_2/He flow for 1 hour and cooled (at 30 °C/min) to 40 °C under He and kept at constant temperature for 30 minutes. After the reduction, doses of CO (10% CO balance He) were introduced into the sample tube at a temperature of 40 °C. Following the completion of the CO pulse test, He was flown through the sample for 20 minutes. The following equation (Eq S4) was used to calculate the % dispersion of the Pd atoms, while Table S3 depicts the values used in the equation for each sample:

$$\%D = \left(\frac{\frac{Q}{S}}{\frac{WtS\%}{WtA}}\right) * 100\% \quad \text{(S4)}$$

Equation S3 was also used in combination with the reported particle size value for comparison.

S1.2.4 X-Ray Adsorption Spectroscopy (XAS)

X-ray absorption spectroscopy data was collected at Inner Shell Spectroscopy beamline (ISS, 8-ID) of the National Synchrotron Light Source (NSLS II) at the Brookhaven National Laboratory. The samples were pressed into wafers by diluting the catalysts with polyethylene glycol in a 1:10 ratio. Passivated Implanted Planar Silicon (PIPS) detector was used to collect fluorescence data. The energy was calibrated using a Pd foil, measured together with the samples, and the edge for metal Pd was set to 24350 kV. To process the data, the Demeter package was used. Between 9 to 16 scans were collected and averaged for each sample to improve data quality. For data analysis, a normalization order of 2 was used with a normalization range between 150.00 to 430.00. The spline range in k ranged from 0 to 11.8 while the spline range in E ranged from 0 to 530. For the forward Fourier transform parameters, a k-range of 3.0 to 9.8 was used while backward Fourier transform parameters had an R-range of 1 to 3.

S1.2.5 X-Ray Diffraction (XRD)

XRD was performed using a Panalytical X'Pert PRO Alpha-1 diffraction system. A CuKα x-ray source was used at a wavelength of $\lambda = 1.54$ Å. For the incident optics, the divergence slit was set to be ½^o while the anti-scatter slit was set to 1°. A 10 mm mask was used with a soller slit of 0.04 radians and a receiving anti-scatter slit of 5.5 mm. A current of 40 mV was applied while the generator operated at a voltage of 45 kV. The spectra were measured from $2\theta = 20^o$ to 90° with a step size of 0.0167^o and a scan speed was 0.0711^o per second. Data analysis and Rietveld refinement was conducted using the HighScore Plus program.

2S1.2.6 N_2 Physisorption

Surface area, pore volume, particle size and pore diameter were measured using N_2 physisorption coupled with Brunauer–Emmett–Teller (BET) analysis. The analysis was conducted on a Micromeritics ASAP 2020 Accelerated Surface Area and Porosimetry System. Approximately 0.200 g of each sample was used during the analysis process. The sample was degassed to 10 mmHg and held under vacuum for 120 mins before backfilling occurred and the sample was shifted to the analysis port where a standard N_2 physisorption method was used to complete the desorption.

Section S1.3: Characterization of Support and Product Interactions

S1.3.1 Pyridine Adsorption followed by IR Spectroscopy

Temperature-programmed desorption of pyridine followed by IR spectroscopy was used to measure the Lewis acidity of the potential catalysts. The catalyst powders were hydraulically pressed into thin wafers and placed in a high-vacuum chamber. A Thermo Scientific Nicolet iS10 spectrometer was used to observe pyridine desorption from catalyst wafers within the high-vacuum chamber, and data was analyzed using the Thermo Scientific Omnic software. Catalyst wafers were activated at 450 °C for 1 h and allowed to cool to 150°C prior to dosing. Pyridine vapor was repeatedly introduced until the pressure equilibrated at ~0.1 mbar, indicating that the natural coverage of Lewis acid sites had reached a maximum. The chamber was evacuated and then heated from 150°C to 250°C, 350°C, and 450°C with cooling in between each temperature setpoint. The Omnic software took scans every five minutes over the 330-minute program, but the meaningful scans occur after the temperature ramps when the chamber returns to 150°C. The strength of acid sites was evaluated based on the temperature at which they can retain pyridine under high-vacuum conditions. The concentration of Lewis acid sites was calculated with the equation S5. Here, S is the ¼" circular area of the catalyst wafer probed by IR spectroscopy, A is the integral of the 1440 cm^{-1} band resulting from pyridine adsorbed on γ-Al_2O_3 Lewis acid sites, and ϵ is the extinction coefficient of 1.71 cm/μmol.

$$C_{LA}(\mu mol * gcat^{-1}) = \frac{S*A}{\epsilon*W} \tag{S5}$$

S1.3.2 Thermogravimetric Analysis (TGA)

TGA was completed using a SDT Q600 V20.9 Build 20 (Figure S6). 15 mg of the catalyst/feedstock mixture post-milled was heated to 550°C at a rate of 10°C per minute under air for Pd_{04}/Al_2O_3 and Pd_{04}/SiO_2.

S1.3.4 Fourier Transform IR Spectroscopy with ATR Attachment

The IR spectra for the solid catalytic mixture pre- and post-reaction were measured using a Thermo Scientific™ Nicolet™ 8700 FTIR spectrometer fitted with Smart iTR™ Attenuated Total Reflectance (ATR) sample loading unit with a diamond/ZnSe crystal lens. A few mg of each

sample was placed over the lens and was held in place using the clamp on the ATR stage. The absorbance spectra were measured from 700 cm^{-1} to 4000 cm^{-1}; 64 scans were taken for each sample, while a fresh background was recorded before each set of sample scans. FTIR was sued exclusively for Pd_{04}/SiO_2 and Pd_{04}/Al_2O_3 samples and BPE and Phenol standards.

Section S1.4: Characterization of Lignin and Resulting Depolymerization

S1.4.1 HSQC NMR

All 1D proton and 2D H1-C13 HSQC spectra were collected under 25C on Bruker Avance III HD 700 MHz spectrometer equipped with TCI cryoprobe. For 1D proton spectra, Bruker standard zg30 pulse sequence was used with recycle delay time d1 = 1 sec and number of scans = 8. For 2D H1-C13 HSQC spectra, Bruker standard hsqcetgpsisp2.2 pulse sequence was used with recycle delay time d1 = 1 sec and number of scans =8; 2048x512 of F2/F1 time domain total points were used; the spectra widths for F2 and F1 dimensions were used as 13 ppm and 220 ppm, and the centers of the spectra were set with O1p = 6.5 ppm and O2p = 90 ppm, respectively. The total NMR time for each 2D spectra was about 1.5 hrs.

Section S2: Supplemental Results

S2.1 Control Experiments

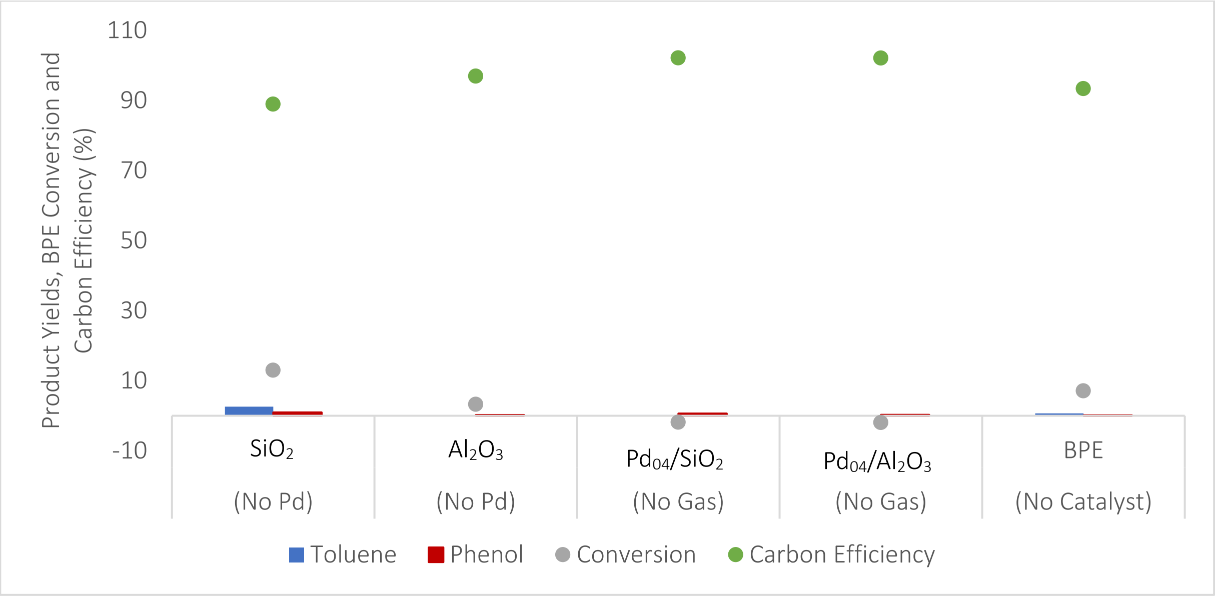

Figure S2: Results for control experiments in the absence of either Pd or H_2. Toluene and phenol yields are shown along with BPE conversion and carbon efficiency.

The control experiments were performed to confirm that both Pd and H_2 must be present to drive this hydrogenolysis reaction and that there was no reactivity if either was not present (Figure S0). The first set of experiments were conducted in the absence of Pd, with only SiO_2 or Al_2O_3 (0.25 g), combined with 0.2 g of BPE. All other variables were held constant: 15 min under 15 sccm N_2 at 3 Hz, 15 min under 15 sccm H_2 at 3 Hz, 60 min under 15 sccm H_2 at 20 Hz, followed by a stagnant 30 min N_2 purge. The results showed minimal product yield for toluene and phenol using silica (2.6% and 1.0%) and no significant product formation for alumina. Next, the Pd supported catalysts were reacted with BPE in a closed vessel, without gas flow. For consistency, the vessels were milled for 30 min at 3 Hz then for 60 min at 20 Hz to match the original milling durations. No significant product yield above 1% was observed for any of these reactions, confirming that H_2 must be present for a significant reaction to occur. Finally, BPE was milled without any catalyst present to test whether the pure mechanical forces of the mill were sufficient enough to drive any type of reaction. Product yields of less than 1% were observed for toluene and phenol, which could aid in explaining why minimal conversion was observed in the previous control experiments. Even without a catalyst or H_2 present, the shear and impact forces of the

collision between the balls and vessel walls consistently resulted in BPE cleavage to a small but measurable extent in most experiments.

The greatest inconsistency was observed for BPE conversion which range from -2% to 13%. In an ideal reaction, the conversion should corresponds to the product yields, but this was not observed because these products were sticky and extremely difficult to collect. A combination of dry-scraping and methanol washing was used to collect what was possible from the mill, but the physical inability to recover every particle within the vessel resulted in what appeared to be conversion of BPE when the extent of reaction was actually an error resulting from experimental limitations. Likewise, small negative conversion values can be attributed to systematic or instrumental errors. In all of these cases, the carbon balance was directly reflected by the BPE conversion value; if full collection was possible, the carbon balance was close to almost 100%.

S2.2 Turn over Frequency (TOF) Calculations

To calculate the TOF for each catalyst, the g of metal in each catalyst sample (Eq S6) was first calculated using the metal loadings found via ICP testing.

$$Moles\ of\ Metal = \frac{Metal\ wt\ \% * Total\ g\ of\ catalyst}{MW\ of\ metal} \tag{S6}$$

Next, average toluene yield per minute was calculated by subtracting the initial toluene yield at 0 min from the yield at 30 mins for Pd-based catalysts and 60 mins for the Ni-Based catalyst (Eq S7). At 0 min, the Ni catalysts had a toluene yield of 0%, but Pd_{04}/Al_2O_3 and Pd_{04}/SiO_2 had yields of 8% and 6% respectively. As previously mentioned, the mill was purged for 15 min under H_2 at a low frequency (3 Hz) before heavy milling at 20 Hz; this non-zero yield before heavy-impact milling reiterates how much more reactive Pd is in comparison to Ni, which is reflected in the final TOF values (Eq S8). Final values are shown in Figure S1.

$$Toluene\ yield\ per\ minute = \left(\frac{Total\ toluene\ \% * Moles\ BPE}{100} - \frac{Initial\ toluene\ \% * Moles\ BPE}{100}\right) \Big/ Milling\ Minutes \tag{S7}$$

$$TOF\ in\ min^{-1} = \frac{Toluene\ yield\ per\ minute}{Moles\ of\ metal} \quad (S8)$$

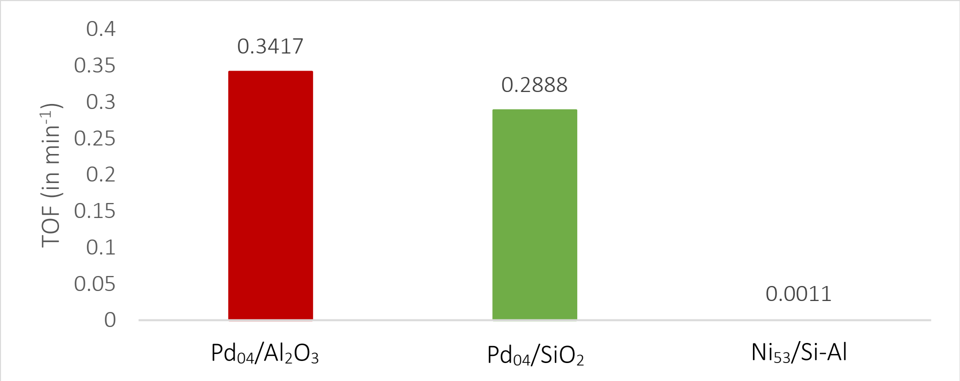

Figure S3: TOF values in min^{-1} for Pd_{04}/Al_2O_3, Pd_{04}/SiO_2 and Ni_{53}/Si-Al calculated using equations 1-3 above.

S2.3 Inductively Coupled Plasma (ICP) Results

Table S1: ICP results for the three catalysts before milling. For rounding simplicity, all Pd wt % values were denoted as having 4% in abbreviation descriptions.

Support	*SiO_2*	*Al_2O_3*
Pd Content (mg/kg)	*38398*	*36220*
Pd Weight %	*3.84%*	*3.62%*

S2.4 Transmission Electron Microscopy (TEM) Images and Dispersion Values

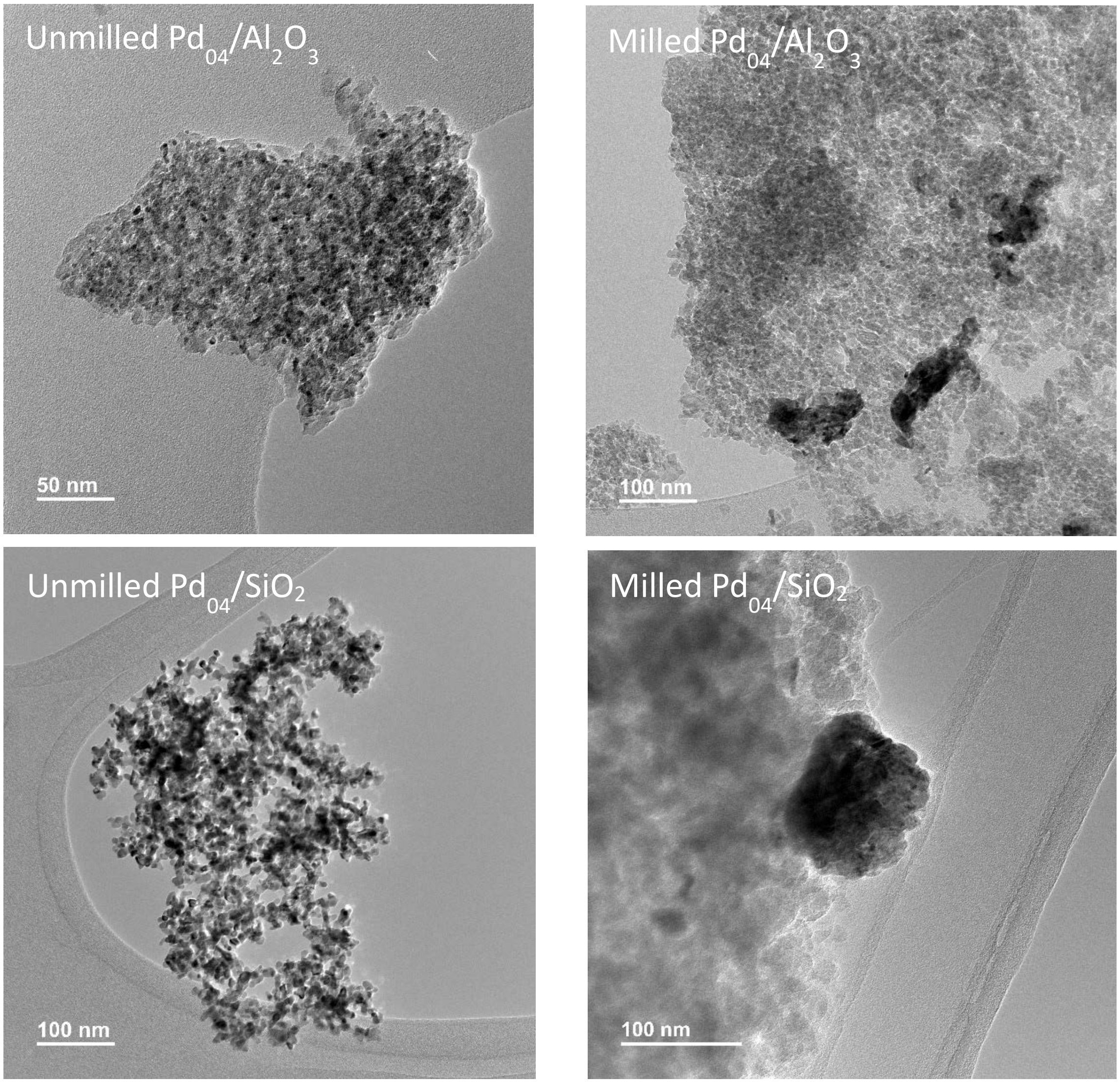

Figure S4: TEM images for milled and unmilled Pd_{04}/Al_2O_3 and Pd_{04}/SiO_2. Particle agglomeration and reduced Pd surface area exposure is evident post-milling. Scales shown at either 50 or 100 nm.

Table S2 depicts the values used to quantify D% (Eq S3) based off the measured average diameter of the particles from the above TEM images in Figure S2.

Table S2: Dispersion values using equation S3 based on particle sizes found via TEM imaging.

	Pd_{04}/Al_2O_3	Pd_{04}/SiO_2
Particle Size (nm)	3.472	7.289
Dispersion (%)	32.25	15.36

S2.5 CO Chemisorption Values

Table S3: Values provided by the Mircomeritics AutoChem II 2920 Analysis used for %D calculations using equation S4.

	Pd_{04}/Al_2O_3	Pd_{04}/SiO_2
Q = Quantity Absorbed	154.92147 µmol/g	85.57415 µmol/g
S = Stoichiometric Factor	1	1
WtS% = % Wt Pd in Sample	3.62%	3.84%
WtA = Atomic Weight	106.4 g/mol	106.4 g/mol
Dispersion	45.53 %	23.71 %

Table S4: Dispersion values using equation S3 based on particle sizes from CO chemisorption.

	Pd_{04}/Al_2O_3	Pd_{04}/SiO_2
Particle Size (nm)	2.459	4.724
Dispersion (%)	45.53	23.70

As stated above, two methods were utilized to analyze particle size and dispersion. TEM and CO chemisorption were used with equation S3 to demonstrate the possible dispersion range, while CO chemisorption was used with equations S3 and S4 to demonstrate that both analytical equations produce the same dispersion values. It is known that these equations imply simple assumptions based on a spherical particle shape, when actual dispersion values can be complex and vary by particle.[3, 4] For this reason, we see a significant range between the two values, roughly 12%, rather than direct correlations with minimal error between these two methods.

S2.6 X-Ray Adsorption Spectroscopy (XAS) Spectra for the Pd edge

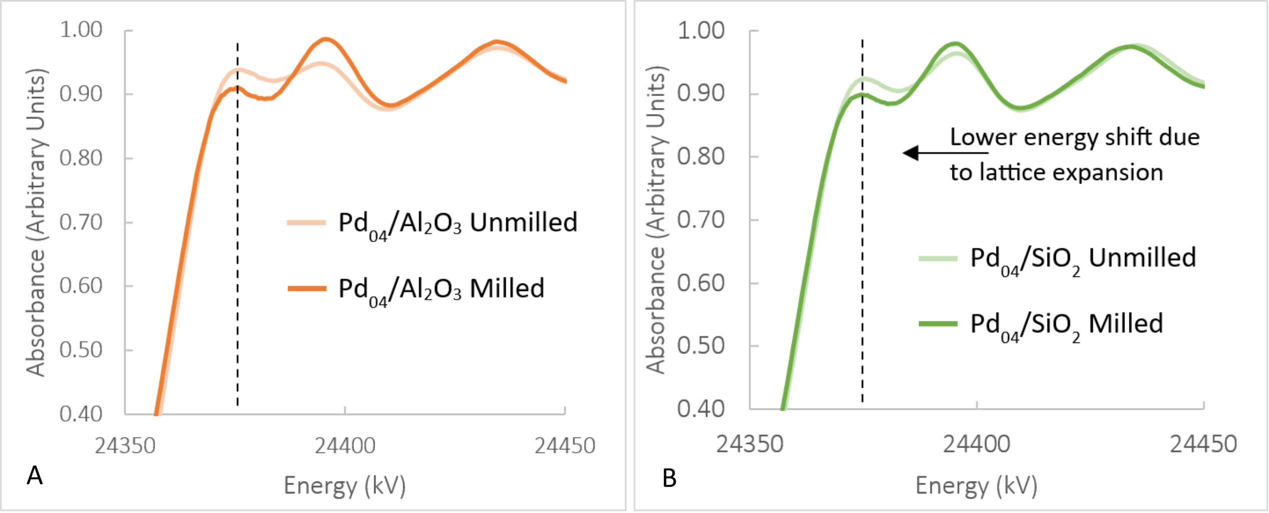

Figure S5: XANES spectra for Pd_{04}/Al_2O_3 (A) and Pd_{04}/SiO_2 (B) catalysts before and after milling to depict hydride formation which is indicated by lattice expansion and a lower energy shift. This indicates a reduction in the oxidation state of Pd.

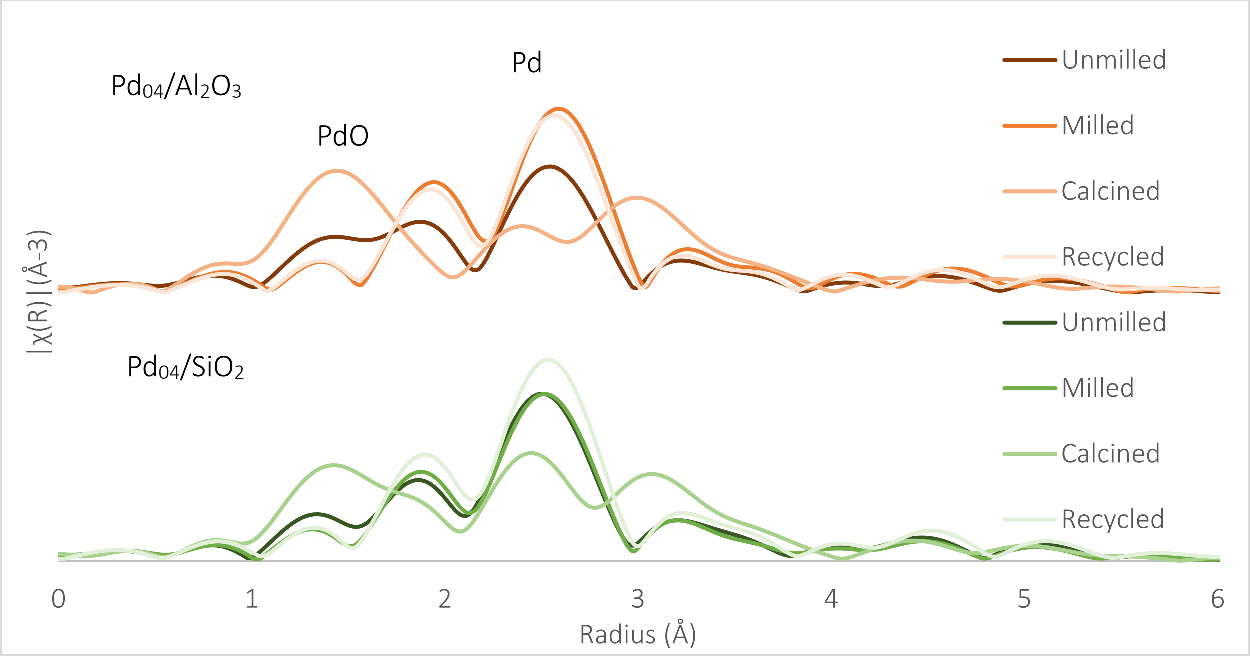

Figure S6: Fourier transformed R-space of the experimental Pd K-edge EXAFS signals for Pd_{04}/Al_2O_3 and Pd_{04}/SiO_2 catalysts before and after milling, after calcination and after recyclability testing.

S2.7 Pyridine Adsorption followed by IR Spectroscopy

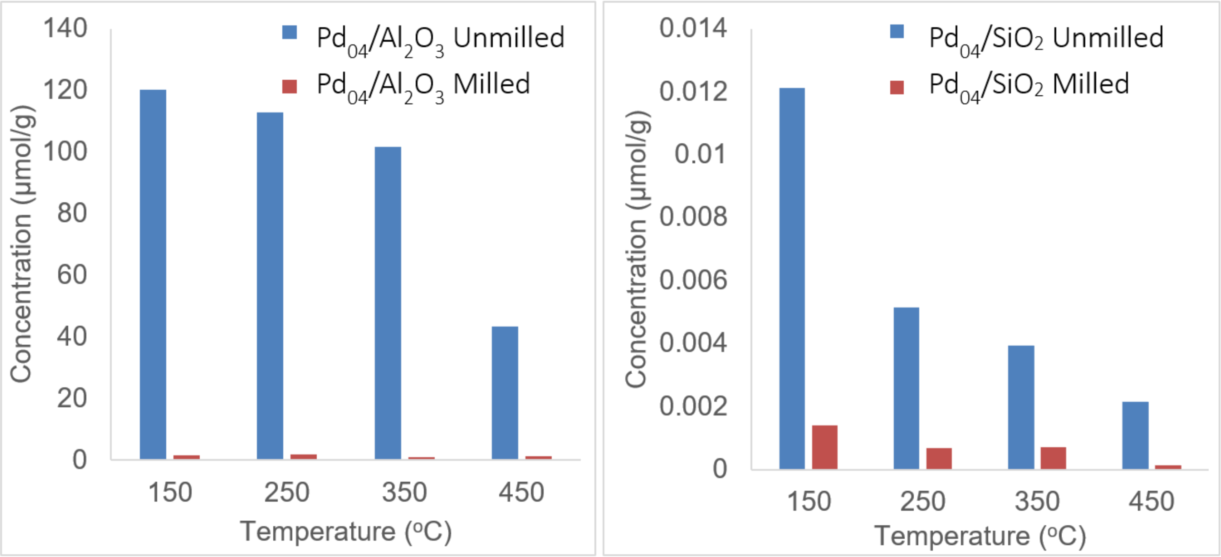

Figure S7: Lewis acid site concentrations for Pd_{04}/Al_2O_3 and Pd_{04}/SiO_2 catalysts before and after milling calculated using pyridine adsorption followed by IR spectroscopy analysis.

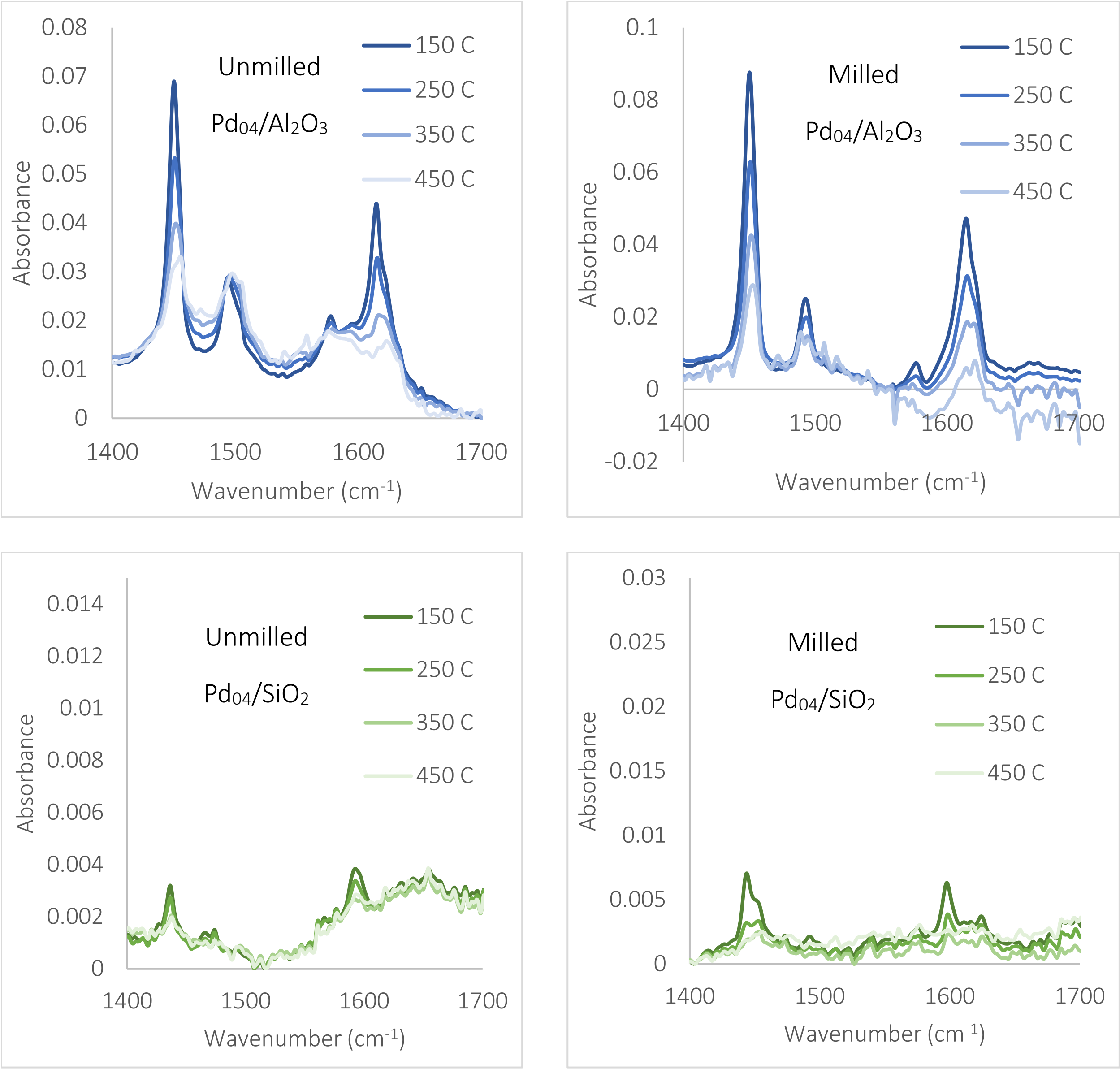

Figure S8: FTIR spectra for Pd_{04}/Al_2O_3 and Pd_{04}/SiO_2 catalysts before and after milling using pyridine adsorption follow by heating from 150°C to 250°C, 350°C, and 450°C with cooling in between each temperature to observe the decrease in Lewis acid cite concentration at 1440 cm^{-1}.

Lewis Acid Site (Pd_{04}/Al_2O_3) and Phenolic Calculations:

LA Sites determined by Pyridine Adsorption: 120.1µmol/g fresh catalyst

1.5 µmol/g spent catalyst

➔ 0.25 g catalyst used per trial = 30.025 µmol of LA sites present

$$\frac{0.2\ g\ BPE}{1} * \frac{1\ mol\ BPE}{184.23\ g\ BPE} * \frac{1\ mol\ phenol}{1\ mol\ BPE} * \frac{94.11\ g\ phenol}{1\ mol\ phenol}$$

$$= 0.1021\ g\ Phenol\ theoretical\ yield$$

Average actual phenol yield = 48% → 0.0490 g phenol

Unaccounted for phenol = 52% → 0.0531 g phenol

$$\frac{0.0531\ g\ phenol}{1} * \frac{1\ mol\ phenol}{94.11\ g\ phenol} * \frac{10^6 \mu mol\ phenol}{1\ mol\ phenol} = 564.2\ \mu mol\ phenol$$

While roughly 30.03 µmol of Lewis acid sites are present on the Pd_{04}/Al_2O_3 catalyst initially, it is known that additional defect sites are created with milling that can lead to phenol adsorption. As hydrogenolysis occurs in tandem with the creation of defect sites, phenol adsorbs to the open sites and resists further hydrogenation to cyclohexanol because it is stable once adsorbed. This is confirmed through FTIR conducted with an ATR attachment which shows strong aromatic presence after milling, and minimal cyclohexane peaks. As shown above, there are 564.2 µmol of phenol unaccounted for on average for both Pd_{04}/Al_2O_3 and Pd_{04}/SiO_2, which suggests that the Lewis acid sites have minimal impact on absorption in comparison to defect sites. LA site occupation accounts for ~5.3% (30/564) of missing phenolics with Pd_{04}/Al_2O_3; by comparison, defect sites account for ~94.7% (534/564). It was determined that created silanol sites and defect sites through milling Pd_{04}/SiO_2 adsorbed all missing phenolic products, considering Pd_{04}/SiO_2 LA site concentration was initially a minimal 0.012 µmol/g.

S2.8 Thermogravimetric Analysis (TGA) Results

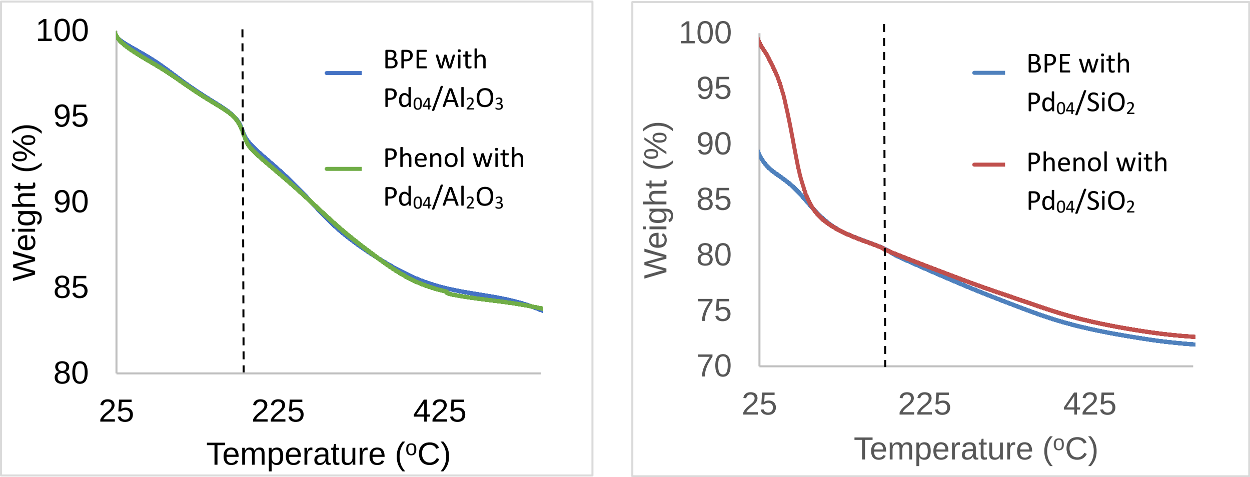

Figure S9: TGA spectra of organic residue weight loss. BPE milled with Pd_{04}/Al_2O_3 and Pd_{04}/SiO_2 is shown, along with phenol milled with Pd_{04}/Al_2O_3 and Pd_{04}/SiO_2.

To further confirm that phenol was not lost during the milling process, TGA analysis was conducted. First, Pd_{04}/Al_2O_3 and Pd_{04}/SiO_2 were analyzed after milling with BPE under the standard reaction conditions previously stated. For comparison, an equimolar amount of phenol (0.001086 mol) was separately milled with 0.25 g of Pd_{04}/Al_2O_3 and Pd_{04}/SiO_2; this was representative of the amount of phenol that would have been produced as a result of 100% BPE cleavage via hydrogenolysis. Phenol has a BP of 182°C, so it was presumed that any weight loss before was a result of moisture loss or from minimal amount of cyclohexanol product. After 182°C, the curves for Pd_{04}/Al_2O_3 are aligned while the curves for Pd_{04}/SiO_2 were nearly aligned; the curve for BPE milling with Pd/SiO_2 shows an additional 0.7% weight loss. The rate of weight loss indicated that phenol was being burned off both samples at the same pace. This confirms the previously established GC-FID results that phenol was the main product, and no coking occurs, and that the full amount of hypothetical phenol is present post reaction. Therefore, the ‘missing’ 52% phenol yield must be a result of chemisorbed or physisorbed phenolic interaction with the support rather than any side repolymerization or system leakage.

2.9 XRD of the Al_2O_3 Support

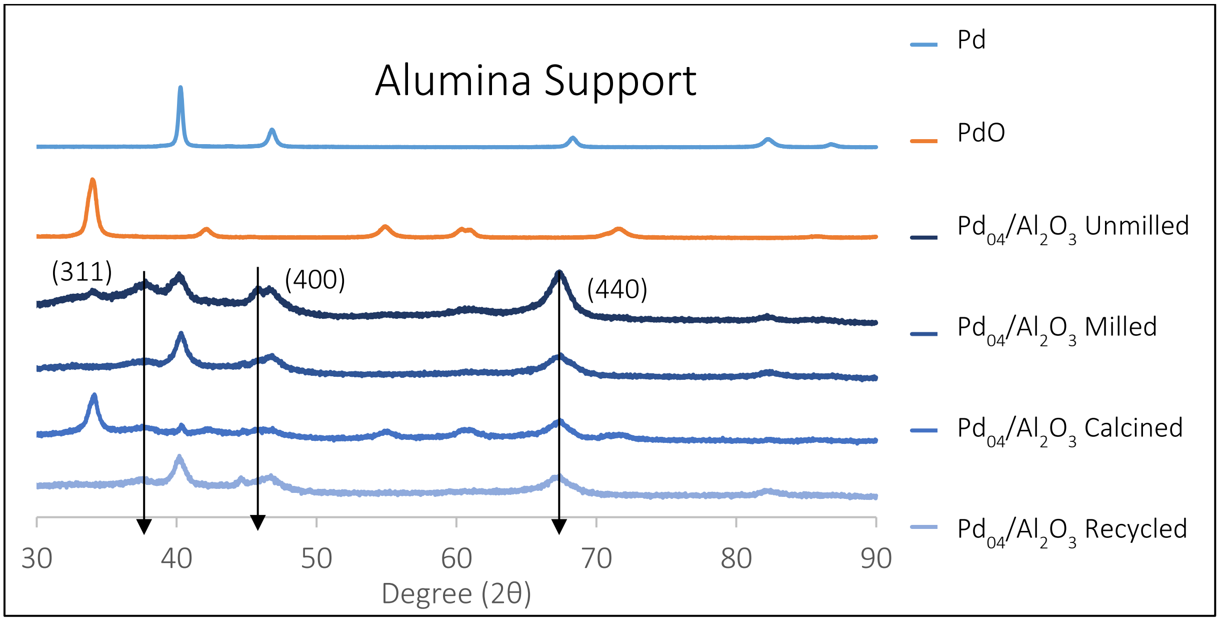

Figure S10: XRD spectra of Pd_{04}/Al_2O_3 throughout the milling and recycling process between 30° to 90° with the parent peaks for Al_2O_3 highlighted at 38°, 46° and 68°.

XRD indicated a decrease in crystallinity of the alumina support as a result of the milling process. The two peaks representing to the (311) and (400) diffractions became nearly negligible, while the main peak of the (440) diffraction remained observable but broadened suggesting amorphization.

2.10 FTIR Spectroscopy

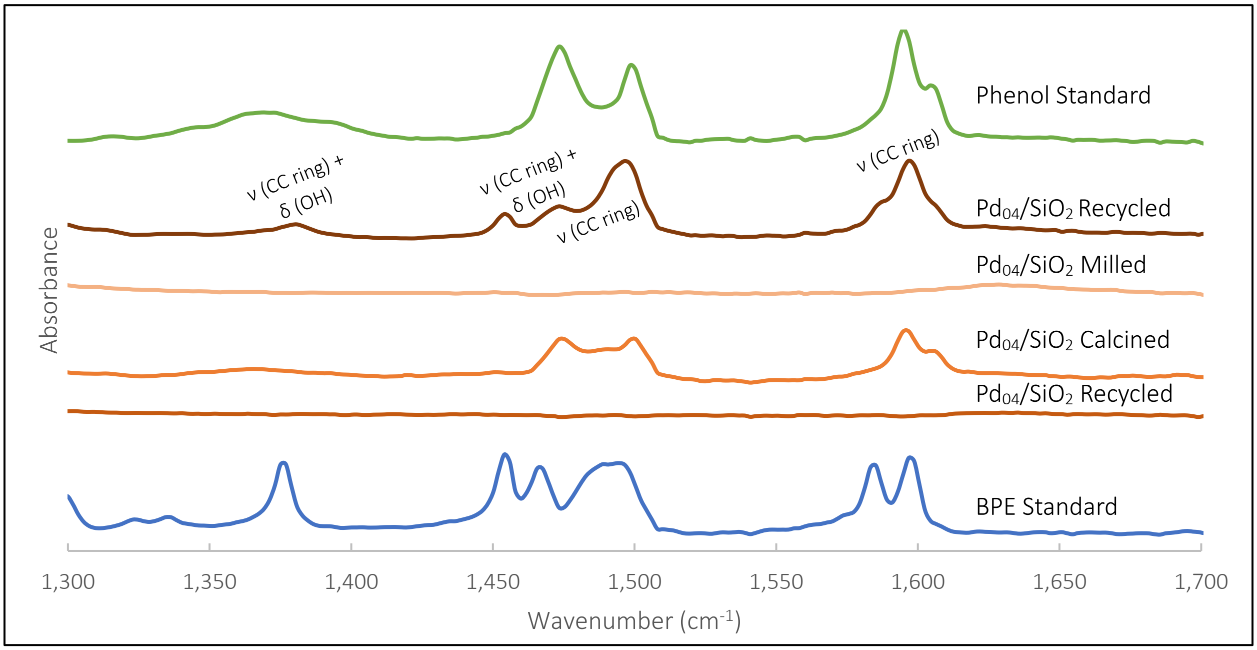

Figure S11: FTIR spectra of Pd_{04}/SiO_2 unmilled, milled, calcined, and recycled.

FTIR spectroscopy was used to study residual organic species on the silica support. In the spectrum of solid phenol, a characteristic peak of aromatic ring vibrations was observed at 1597 cm^{-1} with a small shoulder at 1606 cm^{-1}.[5] The peak at 1500 cm^{-1} is also characteristic of an aromatic ring vibration, while the one at 1477 cm^{-1} and the broad feature around 1383 cm^{-1} correspond to a combinations of δ(OH) and ν(C=C) vibrations.[5] No band were observed between 1300 and 1700 cm^{-1} for fresh Pd_{04}/SiO_2. The FTIR spectrum of the milled catalysts resembled that of phenol, but the features with involving contributions from δ(OH) vibrations were less pronounced. This indicates the presence of chemisorbed phenolate species on the surface. All of these deposits were removed during calcination. The spectrum of the recycled catalyst contained additional characteristic peak that were also observed in the spectrum of pure BPE: a peak at 1456 cm^{-1} corresponding the δ(CH_2) vibration next to the ether group and a peak at 1375 cm^{-1} that is assigned to a ν(C=C) mode with contribution from other vibrations.[6] The presence of these bands indicates the presence of some unconverted BPE in addition to chemisorbed phenolates.

S2.11 HSQC NMR - Unmilled Supercritical Water Extracted Lignin and Milled Lignin with Pd_{04}/SiO_2 and Pd_{04}/Al_2O_3

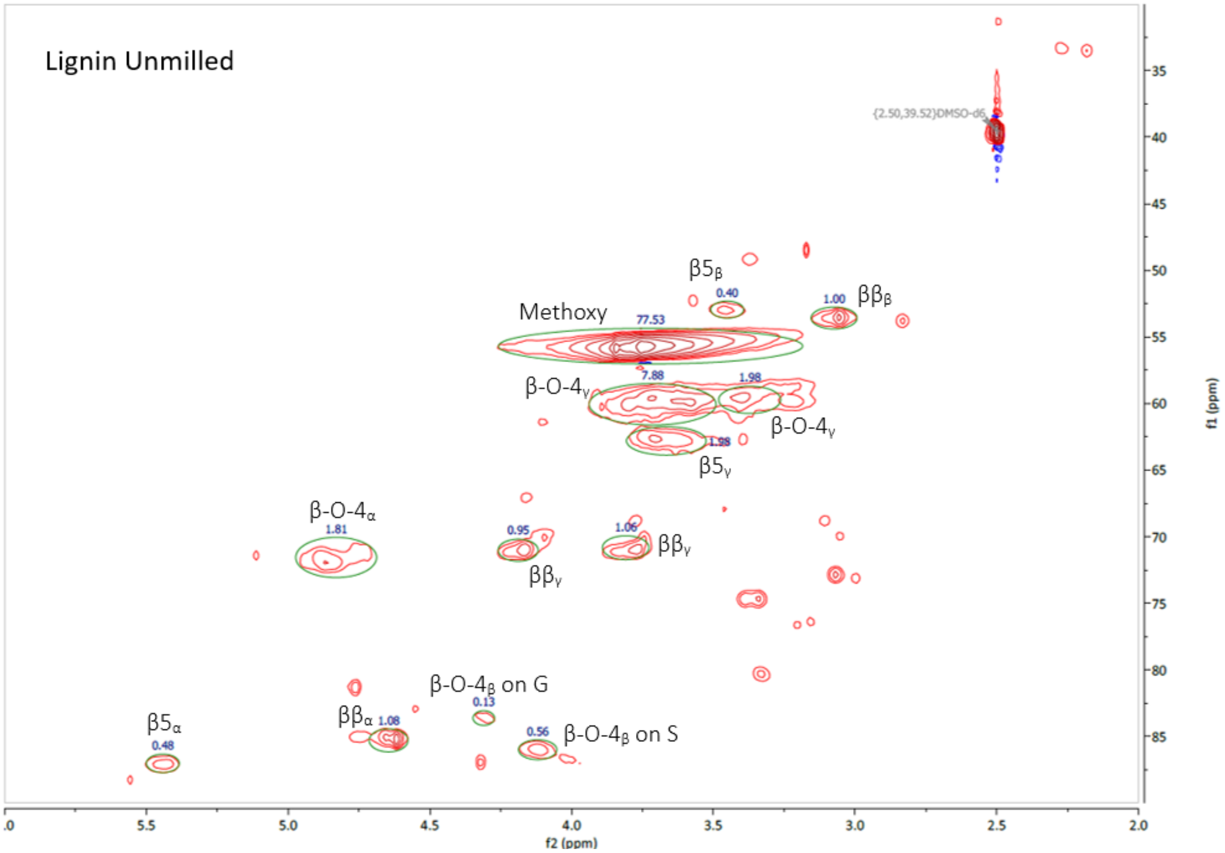

Figure S12: HSQC NMR spectra for unmilled supercritical water extracted lignin with DMSO – d_6 as a reference standard and corresponding integral area values for the ββ, β-5, and β-O-4 bonds.

It is important to note that a slight heterogeneity is typically present in lignin samples, and the abundance of specifics bonds can vary by 3-5 percentage points.[7-10] For this reason, the NMR peak integrals of some bonds, such as the β-O-4$_\alpha$ and the ββ$_\gamma$, appear to slightly increase while other, such as those of the β-O-4$_\beta$ and the ββ$_\alpha$ bonds, decrease. The lack of perfect homogeneity throughout processed lignin samples is a common occurrence and it can therefore be inferred that the unmilled sample above (Figure S13) did not have the exact same initial linkage distribution as the 0.2 g used for the Pd_{04}/Al_2O_3 or the Pd_{04}/SiO_2 milling experiments.

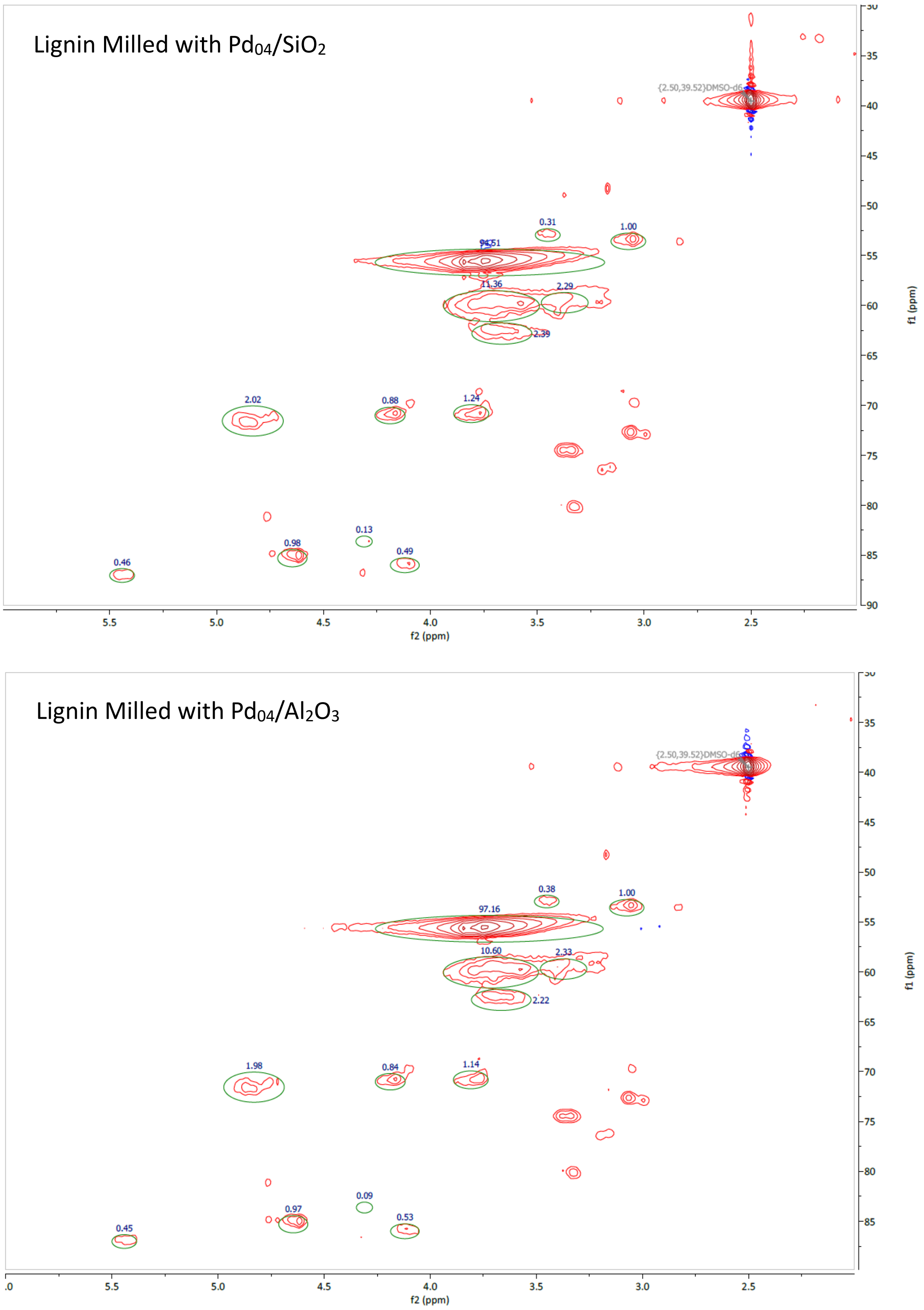

Figure S13: HSQC NMR spectra for supercritical water extracted lignin milled for 1 hr using Pd_{04}/SiO_2 and Pd_{04}/Al_2O_3 with DMSO – d_6 as a reference standard for the corresponding integrals areas.

References:

(1) Reichle, S.; Felderhoff, M.; Schuth, F. Mechanocatalytic Room-Temperature Synthesis of Ammonia from Its Elements Down to Atmospheric Pressure. *Angew Chem Int Ed Engl* **2021**, *60* (50), 26385-26389. DOI: 10.1002/anie.202112095 From NLM PubMed-not-MEDLINE.
(2) Ravel, B.; Newville, M. ATHENA, ARTEMIS, HEPHAESTUS: data analysis for X-ray absorption spectroscopy using IFEFFIT. *J Synchrotron Radiat* **2005**, *12* (Pt 4), 537-541. DOI: 10.1107/S0909049505012719.
(3) OLGA-LETICIA PEREZ, D. R., MIGUEL JOSE YACAMAN. The Relation between Dispersion and Particle Size on Supported Catalysts. *Journal of Catalysis* **1983**, *79*, 240-241.
(4) Bonarowska, A. B. s. a. M. Relation between Crystallite Size and Dispersion on Supported Metal Catalysts. *Langmuir* **1997**, *13*, 5613-5620.
(5) Popov, A.; Kondratieva, E.; Gilson, J.-P.; Mariey, L.; Travert, A.; Maugé, F. IR study of the interaction of phenol with oxides and sulfided CoMo catalysts for bio-fuel hydrodeoxygenation. *Catalysis Today* **2011**, *172* (1), 132-135. DOI: 10.1016/j.cattod.2011.02.010.
(6) Colthup, N. B. D., L.H.; Wiberley, S.E. *Introduction to Infrared and Raman Spectroscopy*, Third ed.; Academic Press Inc., 1990.
(7) Wang, H.; Tucker, M.; Ji, Y. Recent Development in Chemical Depolymerization of Lignin: A Review. *Journal of Applied Chemistry* **2013**, *2013*, 1-9. DOI: 10.1155/2013/838645.
(8) Li, C.; Zhao, X.; Wang, A.; Huber, G. W.; Zhang, T. Catalytic Transformation of Lignin for the Production of Chemicals and Fuels. *Chem Rev* **2015**, *115* (21), 11559-11624. DOI: 10.1021/acs.chemrev.5b00155.
(9) Cheng, C.; Wang, J.; Shen, D.; Xue, J.; Guan, S.; Gu, S.; Luo, K. H. Catalytic Oxidation of Lignin in Solvent Systems for Production of Renewable Chemicals: A Review. *Polymers (Basel)* **2017**, *9* (6). DOI: 10.3390/polym9060240.
(10) Lu, Y.; Lu, Y.-C.; Hu, H.-Q.; Xie, F.-J.; Wei, X.-Y.; Fan, X. Structural Characterization of Lignin and Its Degradation Products with Spectroscopic Methods. *Journal of Spectroscopy* **2017**, *2017*, 1-15. DOI: 10.1155/2017/8951658.
